# Molecular detection and distribution of *Treponema* species in a commercial dairy cattle herd in Slovakia

**DOI:** 10.1007/s11259-025-10897-4

**Published:** 2025-09-19

**Authors:** Simona Mekková, Miriam Sondorová, Petra Ivančová, Natália Šurín Hudáková, Marián Maďar, Marián Kadaši, Pavol Mudroň

**Affiliations:** 1 Clinic of Ruminants, University Veterinary Hospital, University of Veterinary Medicine and Pharmacy in Košice, Komenského 73, Košice, 041 81 Slovakia; 2 Department of Microbiology and Immunology, University of Veterinary Medicine and Pharmacy in Košice, Komenského 73, Košice, 041 81 Slovakia

**Keywords:** *Treponema* spp., Bovine digital dermatitis, PCR, Dairy cattle herd

## Abstract

We analyzed the occurrence and distribution of selected *Treponema* species in dairy cows using 335 interdigital swabs, 335 fecal samples, and 25 surface swabs from bovine digital dermatitis (BDD) lesions. In surface swabs from BDD lesions, *Treponema medium* (92%), *Treponema pedis* (88%), and *Treponema brennaborense* (56%) were the most frequently detected species. Interdigital swabs from BDD-positive cows revealed the presence of *T. medium* in 40%, *T. pedis* in 12%, and *T. brennaborense* in 4% of samples. In the healthy group, *T. medium* was detected in 21.3%, *T. pedis* in 4.8%, and *T. brennaborense* in 1.3% of samples. *Treponema* species were also detected in fecal samples from both groups, with *T. brennaborense* found in 28% of BDD-positive cows and 22.3% of healthy cows, while *T. pedis* was present at a similar rate (8%) in both groups. In fecal samples from healthy cows, the proportion of *T. brennaborense* was significantly higher compared to *T. medium* and *T. pedis* (*p* < 0.001). Similarly, analysis of interdigital swabs showed a significantly higher occurrence of *T. brennaborense* compared to *T. medium* (*p* < 0.001). These findings highlight distinct distribution patterns of *Treponema* species across different sample types and suggest their potential relevance in the diagnosis and understanding of reservoir dynamics in BDD infections.

## Introduction

Since the first report of bovine digital dermatitis (BDD) in dairy cows in the 1970 s from three different regions of Italy (Cheli and Mortellaro 1974), and its subsequent documentation in the United Kingdom in the 1980 s (Blowey and Sharp 1988), the condition has become globally recognized and studied as a major cause of lameness. Originally characterized as a multifactorial polymicrobial disease in dairy cows, BDD has also been documented in beef cattle, sheep, and more recently in goats (Wilson-Welder et al. 2015) and North American Elk (Clegg et al. 2015). Spirochetal bacteria, particularly species of *Treponema*, are considered the primary pathogens in clinical BDD. A total of 45 *Treponema* species have been identified in BDD lesions (Nally et al. 2019), with the most widespread and abundant species belonging to three phylogroups: *Treponema medium*, *Treponema phagedenis*, and *Treponema pedis* (Evans et al. 2008; Espiritu et al. 2020). Lesions are most commonly present on the hind legs and are associated with lameness, reduced milk production, decreased reproductive performance, and diminished animal welfare (Bruijnis et al. 2012; Cutler et al. 2013; Dolecheck and Bewley 2018). Depending on the geographical region, data suggest that 10–40% of all lameness cases can be specifically attributed to bovine digital dermatitis (Cook 2016). The proportion of herds with BDD lesions has been reported to be 93.6% in Canada (Solano et al. 2016), 97% in Denmark (Oliveira et al. 2017), and 98.8% in Finland (Pirkkalainen et al. 2021). Cross-sectional studies have identified the prevalence of M2-stage lesions at 6% in Canada (Solano et al. 2017) and 5.7% in Finland (Pirkkalainen et al. 2021).

According to Bell et al. (2023), understanding the survival of BDD-associated *Treponema* under on-farm or host conditions is crucial for gaining insight into the viability of infectious reservoirs. This knowledge could also contribute to the more effective design of control strategies. As noted by Dias et al. (2024), an approach capable of distinguishing between viable and non-viable bacteria would allow for a more comprehensive understanding of this dynamic, as well as a better assessment of the role of skin colonization in the transmission and persistence of BDD within the herd. Roelofs et al. (2024) considered national-level prevalence monitoring a fundamental step in efforts to control the disease and maintain cow productivity, as systemic effects negatively impact both reproduction and milk yield (Mellado et al. 2018). According to Robcis et al. (2023), achieving consensus between individual and collective approaches to therapy and management‒addressing both infectious and non-infectious causes of lameness‒is key. Despite global efforts, the etiology and pathogenesis of BDD remain incompletely understood. Temporal analysis reveals increased efforts toward isolation and characterization since 2015, coinciding with the growing recognition of the economic impact of bovine digital dermatitis (Kusza and Bagi 2025).

The aim of this study was to investigate the prevalence of *Treponema* species in a commercial dairy herd. The study focused on their distribution on lesion surfaces, in the interdigital space, and in feces, as these sites may serve as potential sources of infection. A conventional PCR method was used for detection. Additionally, the study aimed to compare the distribution of *Treponema* species between BDD-negative and lesioned cows.

## Materials and methods

### Assessment of bovine digital dermatitis lesions and sample collection

Interdigital skin swabs and fecal samples were collected from a total of 335 Holstein-Friesian dairy cows all from the same herd. Additionally, 25 surface swabs were obtained from bovine digital dermatitis (BDD) lesions in clinically affected animals. All samples were collected in November 2022 from a commercial dairy farm located in Eastern Slovakia. The cows were housed in lying stalls with concrete flooring covered with straw bedding. Routine claw trimming was carried out biannually by professional hoof trimmers. Notably, no footbath treatments were employed on the farm. At the time of sampling, the herd-level prevalence of BDD was 7.8%. Diagnosis of BDD was performed through visual inspection of the plantar and palmar surfaces of the feet. Several lesions were observed extending into the interdigital cleft, occasionally involving the surrounding skin or spreading dorsally toward the dew claws. Lesions were classified following the system described by Döpfer et al. (1997), with modifications by Berry et al. (2012), wherein M0 indicates healthy skin without lesions, M2 corresponds to the classical ulcerative stage, and M4 denotes a chronic, inactive stage typically characterized by hyperkeratosis and tissue proliferation. Lesions were identified and M-scored by the same attending veterinary practitioner with experience in diagnosing BDD.

The sampling procedure was conducted as follows: each cow was restrained in a trimming chute, and prior to sample collection, the limbs‒regardless of the presence or absence of BDD lesions‒were gently rinsed with water to remove superficial debris. To avoid cross-contamination between animals, nitrile gloves were changed between each individual. All samples were collected by a single veterinary practitioner using a standardized protocol. Only one leg was sampled per animal, with priority given to the hind leg in clinically healthy cows. Depending on the study group, either two or three samples per animal were obtained simultaneously, as previously described. Lesion surface samples were collected using sterile nylon flocked swabs (COPAN Diagnostics, Murrieta, CA, USA), which were gently swabbed across the active lesion for approximately 10 s. The interdigital space was sampled in a similar fashion, applying consistent pressure to ensure sufficient material was collected. Fecal samples were obtained directly from the rectum using clean, single-use rectal sleeves and subsequently transferred into sterile 13 mL tubes (Sarstedt AG & Co. KG, Nümbrecht, Germany). All swab and fecal samples were transported on ice and stored at − 20 °C without transport medium until further processing for PCR analysis.

### DNA extraction and standard PCR assay

Genomic DNA was extracted from lesion surface swabs, interdigital swabs, and fecal samples using the Quick-DNA Fecal/Soil Microbe Miniprep Kit (Zymo Research, Irvine, CA, USA), in accordance with the manufacturer’s protocol. The concentration and purity of the isolated DNA were evaluated using a Nanodrop Eight spectrophotometer (Thermo Fisher Scientific, Waltham, MA, USA) by determining absorbance ratios at 260/280 nm and 260/230 nm. DNA purity values ranged from 1.8 to 2.2, indicating acceptable quality for downstream molecular analysis.

DNA amplification was performed using conventional polymerase chain reaction (PCR) in a total reaction volume of 30.6 µL. Each reaction contained 1 µL of DNA template (50 ng), OneTaq^®^ 2X Master Mix with standard buffer (New England Biolabs, Foster City, CA, USA), 0.6 µL of primers (final concentration 33 µM), and molecular-grade water. Each PCR run included a negative control (molecular water) and positive controls comprising *Treponema pedis* DSM 18,691, *Treponema brennaborense* DSM 12,168, and *Treponema denticola* DSM 14,222 (DSMZ, Braunschweig, Germany). Amplifications were carried out using a TProfessional Basic thermal cycler (Biometra GmbH, Göttingen, Germany) with primer sets described by Brandt et al. (2011). Detection of *T. denticola*, *Treponema vincentii*, *T. medium* ssp. *bovis*, and *T. phagedenis* ssp. *vaccae* targeting the *flaB2* gene was performed under the following thermal profile: initial denaturation at 94 °C for 5 min, followed by 45 cycles of denaturation at 94 °C for 30 s, annealing at 63 °C for 30 s, and extension at 72 °C for 40 s, with a final extension step at 72 °C for 5 min. For detection of *T. pedis* (targeting the *flaB* gene) and *T. brennaborense* (targeting the 16 S rRNA gene), the thermal cycling protocol was identical except for the number of cycles (35) and an annealing temperature of 61 °C.

Amplification products were separated by electrophoresis on 2% agarose gels, and visualized using the intercalating dye GelRed^®^ (Biotium, Inc., Hayward, CA, USA) under ultraviolet (UV) illumination at 254 nm, employing a UVT-20 SE transilluminator (Herolab GmbH, Wiesloch, Germany). PCR-positive samples were subsequently submitted for Sanger sequencing (Microsynth, Vienna, Austria). The resulting chromatograms were processed and analyzed using Geneious software version 8.0.5 (Biomatters, Auckland, New Zealand). Sequence similarity was evaluated via the Basic Local Alignment Search Tool (BLASTn) available at https://blast.ncbi.nlm.nih.gov/Blast.cgi (accessed on 19 February 2024).

### Statistical analysis

The Chi-square test, incorporating Yates’ continuity correction, was employed to evaluate the distribution of specific *Treponema* species (*T. pedis*, *T. brennaborense*, and *T. medium*) across different sample types, namely lesion surface swabs, interdigital swabs, and fecal samples. For each bacterial species, detection frequencies were compared among the various sampling methods. The number of positive detections from one sample type was statistically assessed against the corresponding counts from the other sample types. All statistical analyses were conducted using R software (version 4.4.1; R Core Team, Vienna, Austria, 2016). A p-value < 0.05 was considered indicative of statistical significance. Yates’ correction was applied to the Chi-square test to adjust for small expected cell counts in the contingency tables, thereby minimizing the risk of Type I errors.

### Ethical approval

The animal study protocol was approved by the Ethics Committee of the University of Veterinary Medicine and Pharmacy in Kosice under protocol code EKVP/2023-03 for studies involving animals.

## Results

### Molecular screening of the prevalence and distribution of Treponema spp. in the production herd by standard PCR

Swab samples from intact interdigital skin and fecal samples were collected from all dairy cows included in the study (*n* = 335). Among these, 25 animals (7.5%) were clinically diagnosed with bovine digital dermatitis (BDD), comprising 24 individuals with active M2-type lesions and one individual presenting with a chronic M4-type lesion. The remaining 310 cows (92.5%) exhibited no clinical signs of BDD and were classified as clinically healthy.

Swab samples from intact interdigital skin and fecal samples were collected from all dairy cows included in the study (*n* = 335). Among these, 25 animals (7.5%) were clinically diagnosed with bovine digital dermatitis (BDD), comprising 24 individuals with active M2-type lesions and one individual presenting with a chronic M4-type lesion. The remaining 310 cows (92.5%) exhibited no clinical signs of BDD and were classified as clinically healthy.

#### Prevalence of Treponemes in BDD negative cows

Molecular analysis was performed to assess the presence of *Treponema* species in the 310 dairy cows without clinical signs of BDD. *T. brennaborense*, a species frequently linked to BDD, was detected in 22.3% (69/310) of fecal samples and appeared to be associated primarily with possible gastrointestinal origin or contamination. *T. pedis* was identified in 8.4% (26/310) of fecal samples, whereas *T. medium* was not detected in any of the samples analyzed.

In swab samples collected from intact interdigital skin, *T. medium* was the predominant *Treponema* species detected, present in 66 samples (21.3%). *T. pedis* was identified in 15 samples (4.8%), while *T. brennaborense* was detected in 4 samples (1.3%). Furthermore, *Treponema socranskii* subsp. *buccale* was found in 2 samples (0.6%), and *T. phagedenis* subsp. *vaccae* was confirmed in 1 sample (0.3%).

Chi-square analysis of interdigital space swabs and fecal samples revealed a significantly higher prevalence of *T. brennaborense* compared to *T. medium* (*p* < 0.01).

#### Prevalence of Treponemes in BDD positive cows

Molecular analysis of samples from cows clinically positive for BDD (stages M2 and M4) revealed a high prevalence of *T. medium*, detected in 92% of superficial lesion swabs and 40% of interdigital swabs. *T. pedis* was identified in 88% of lesion surface swabs, 12% of interdigital swabs, and 8% of fecal samples. *T. brennaborense*, often associated with gastrointestinal tract contamination, was more frequently detected in surface swabs from BDD lesions (56%) compared to fecal samples (28%) and interdigital swabs (4%). The number of samples analyzed and the proportion of PCR-positive results for each *Treponema* species stratified by lesion stage (M2 and M4) are summarized in Table 1.

**Table 1:** Results of detection of Treponema spp. In samples from BDD positive cows

			*PCR detection*:		
Type of sampling^a^	BDD^b^	Number of samples	***Treponema medium***	***Treponema pedis***	***Treponema brennaborense***
S	M2	24	23 [92]	22 [88]	14 [56]
S	M4	1	0	0	0
ID	M2	24	9 [36]	3 [12]	1 [4]
ID	M4	1	1 [4]	0	0
F	M2	24	0	2 [8]	7 [28]
F	M4	1	0	0	0

^a^ *S* superficial swab from the lesion, *ID* swab from the interdigital space, *F* faecal sample; ^b^ *M2* active form of bovine digital dermatitis, ^b^ *M4* chronic form of bovine digital dermatitis; *[%]* percentage of bovine digital dermatitis

**Fig. 1:**
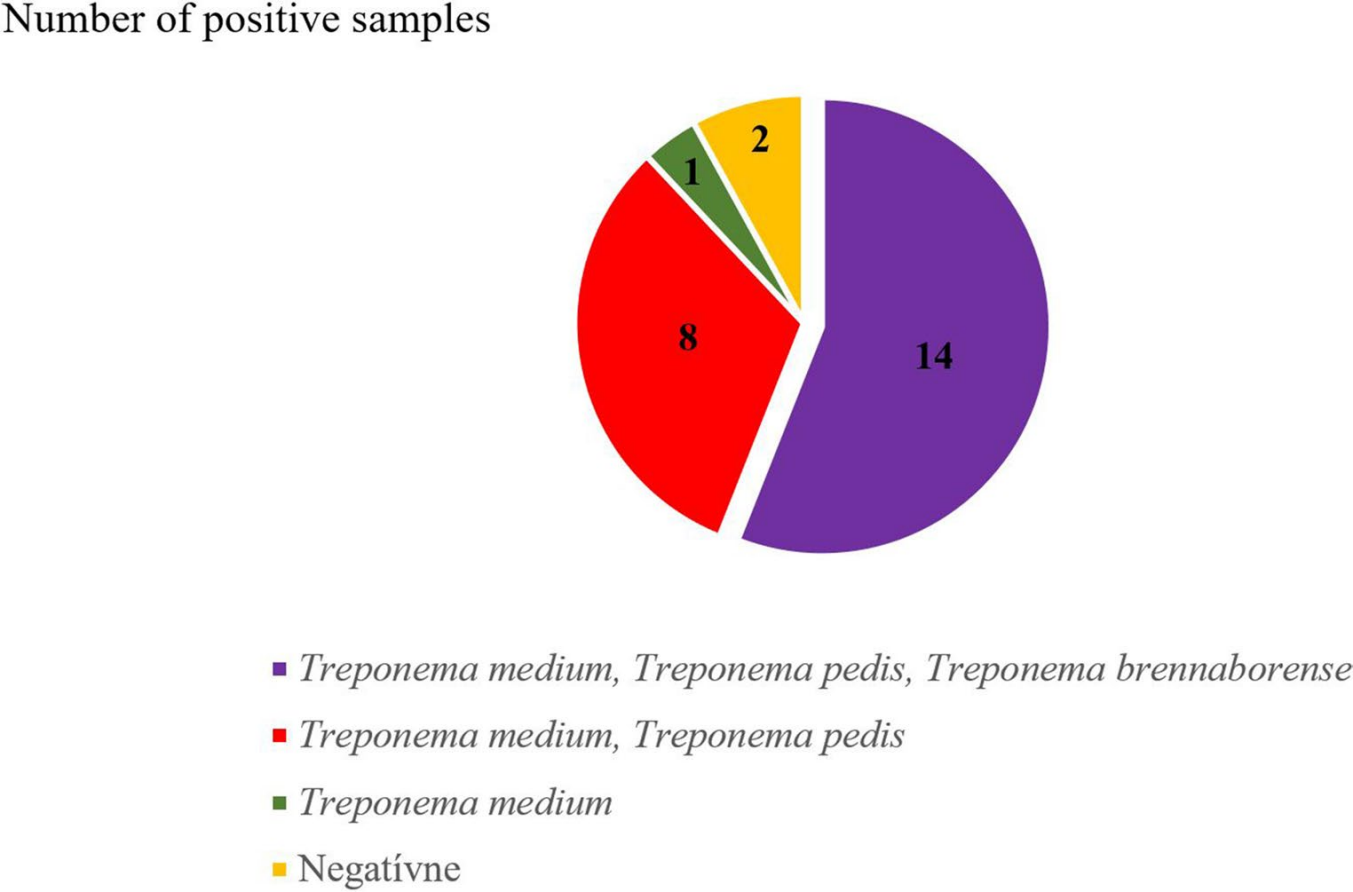
Treponema spp. communities on the surface of BDD lesions

PCR analysis of surface swab samples from BDD lesions demonstrated distinct distribution patterns of *Treponema* species. The concurrent presence of all three targeted species was the most common finding, observed in 56% of samples. Dual-species combinations were detected in 32% of samples, while a single *Treponema* species was identified in 4% of cases (see Fig. 1).

#### Evaluation of Treponemal distribution in cows without a BDD lesion and comparison with cows with a lesion

Molecular analysis of interdigital space samples from clinically healthy cows revealed the presence of *Treponema* spp. DNA in 28.4% of the samples (PCR-positive). Detected species included the three primary targets‒*T. medium*, *T. pedis*, and *T. brennaborense*‒as well as *T. socranskii* subsp. *buccale* and *T. phagedenis* subsp. *vaccae*. In contrast, the prevalence of *Treponema* spp. DNA in swab samples from cows with BDD lesions was significantly higher, reaching 56%. Analysis of fecal samples showed the presence of *Treponema* spp. DNA in 95 out of 310 samples (30.6%) from clinically healthy cows and in 9 out of 25 samples (36%) from cows with BDD; this difference was not statistically significant. Notably, *T. socranskii* subsp. *buccale* and *T. phagedenis* subsp. *vaccae* were not detected in any samples derived from BDD-affected animals.

**Fig. 2:**
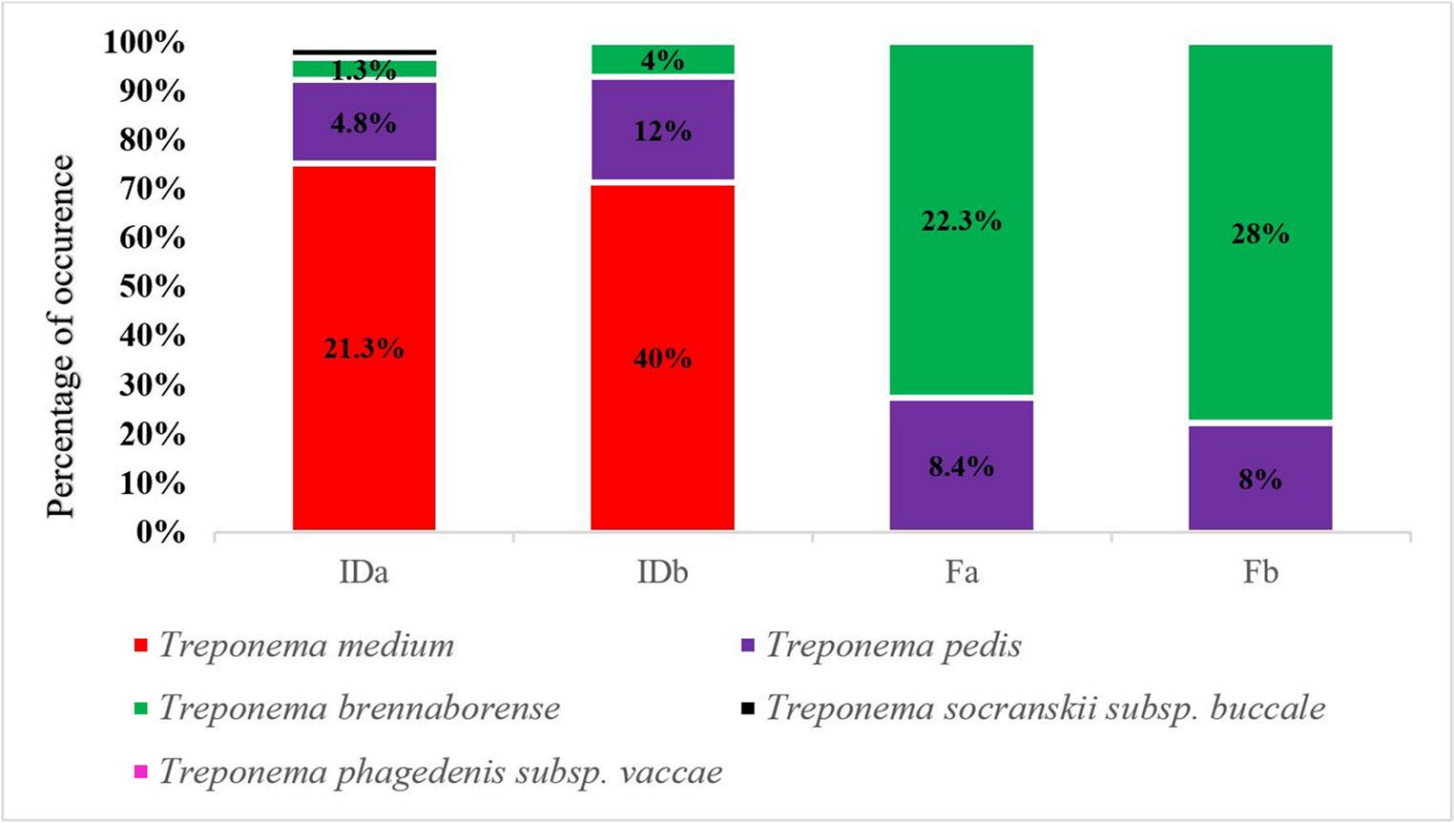
Comparison of the distribution of Treponema spp. by analysis of samples from cows with a BDD lesion and clinically healthy cows, Percentage (%) presence of each Treponema species. IDa - swab from the interdigital space of the claw without BDD lesion; IDb - swab from the interdigital space of the claw with BDD lesion; Fa - faecal sample without BDD lesion; Fb - faecal sample with BDD lesion

Figure 2 presents a comparison of the distribution of *Treponema* spp. in interdigital space swab samples and fecal samples from dairy cows with and without BDD lesions. In interdigital swabs, *T. medium*, *T. pedis*, and *T. brennaborense* were detected more frequently in BDD-affected cows. In contrast, *T. medium* was not detected in any fecal samples. The analysis also revealed only minor differences in the prevalence of PCR-positive detections of *T. pedis* and *T. brennaborense* between fecal samples from clinically healthy cows (without BDD lesions) and those from cows with BDD.

Statistical analysis of the BDD infection reservoir survey results, performed using the Chi-square test with Yates’ correction, revealed a significantly higher prevalence of *T. brennaborense* compared to *T. medium* and *T. pedis* (*p* < 0.001) in fecal samples from healthy cows. Additionally, the difference between *T. pedis* and *T. medium* was also statistically significant (*p* < 0.001). The incidence of *T. brennaborense* detected by interdigital swabbing was significantly higher than that of *T. medium* (*p* < 0.001) and *T. pedis* (*p* < 0.05). Additionally, the difference in prevalence between *T. pedis* and *T. medium* using interdigital swabbing was statistically significant (*p* < 0.001).

**Fig. 3:**
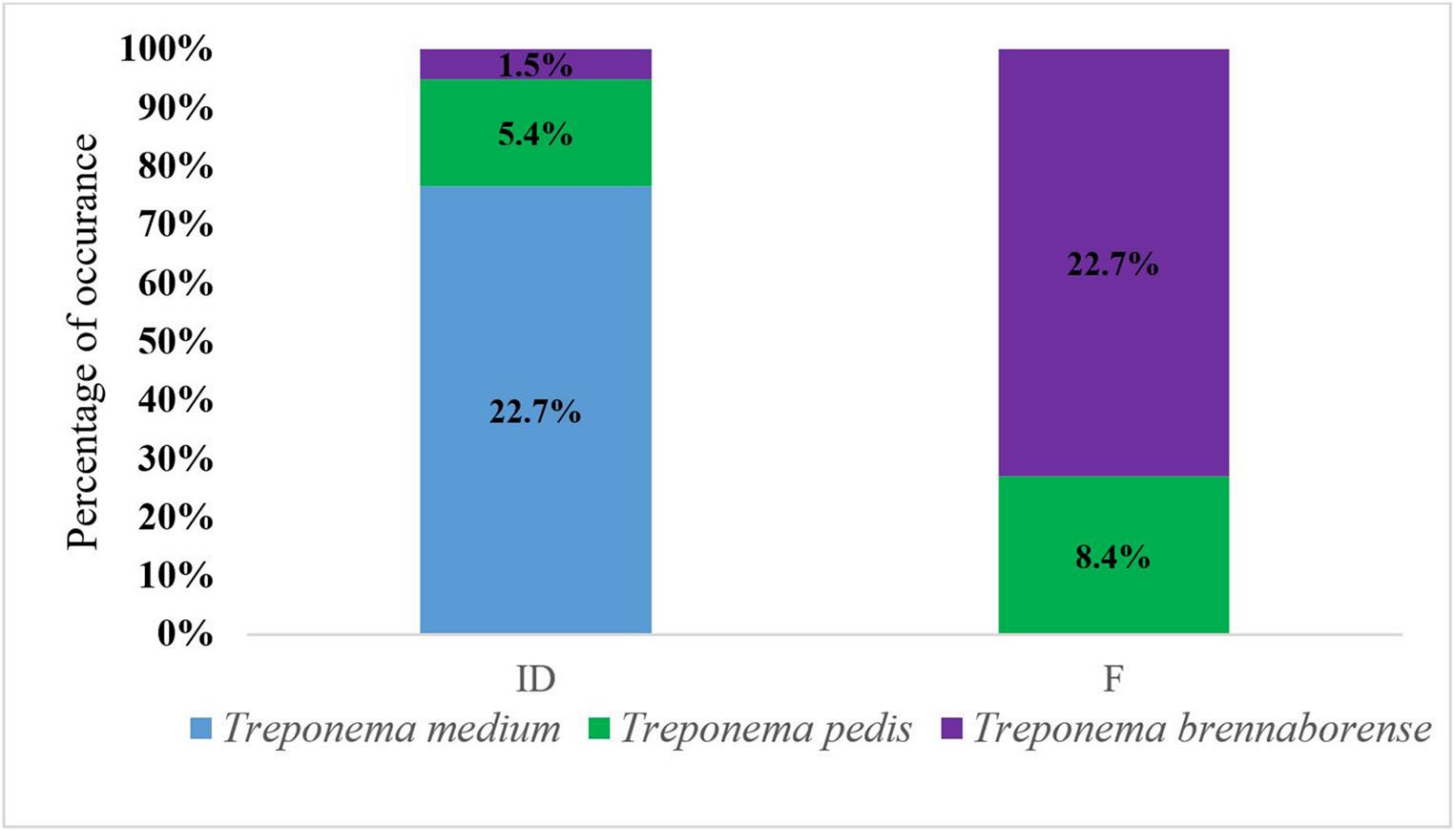
Distribution of Treponema spp. regardless of limb status. ID - interdigital swab; F - faecal sample

The overall prevalence of *Treponema* spp. in interdigital space and fecal samples, regardless of the presence of bovine digital dermatitis lesions (Fig. 3), suggests the potential existence of reservoirs for *Treponema* transmission. Specifically, *T. medium* was detected most frequently in interdigital space samples, whereas *T. brennaborense* showed a higher prevalence in fecal samples.

Sequencing of amplified products from fecal samples using selected primer pairs, followed by BLASTn analysis, revealed an average similarity of 98.66% for *T. pedis* and 97.24% for *T. brennaborense*. For amplified products from interdigital space samples, BLASTn analysis showed an average similarity of 99.53% for *T. medium*, 99.75% for *T. pedis*, and 99.96% for *T. brennaborense*.

## Discussion

*Treponema* spp. have also been detected on the skin of clinically healthy limbs (Caddey et al. 2021; Frosth et al. 2023), although studies involving samples from healthy animals remain limited. A recent study by Dias et al. (2024), which compared bacterial counts on healthy skin and within lesions, suggests that colonization of healthy skin may be transient, likely resulting from environmental contamination. However, the development of BDD appears to be more closely associated with the presence of specific *Treponema* species rather than with general treponemal colonization (Gomez et al. 2012). This finding highlights the need for longitudinal studies aimed at better understanding the colonization dynamics of *Treponema* spp. and determining whether their presence on healthy skin is transient or persistent over time. Such research would probably be highly valuable for elucidating how *Treponema* species‒particularly those associated with BDD‒colonize, persist, and behave at various stages of infection.

In samples from BDD lesions, *Treponema* spp. were identified at a frequency of 87%, with *T. medium* detected in 87.5%, *T. phagedenis*-like in 86.2%, and *T. pedis* in 78.3% of cases (Salem et al. 2025). In the second part of our study, which involved analysis of 25 samples from BDD lesions, *T. medium* was detected in 92% of superficial swab samples, *T. pedis* in 88%, and *T. brennaborense* in 56%. *T. phagedenis* was not detected in any of the samples. In our previous study (Mekková et al. 2025), we analyzed the distribution of *Treponema* spp. in tissue samples, surface swabs, and fecal samples from 20 cows with M2-stage BDD lesions, and compared detection methods using conventional PCR and real-time PCR. Krull et al. (2014) reported a significant increase in the relative abundance of *Treponema* spp., from 1.3% in control samples to 69% in lesions classified as initiated and progressive, with *T. phagedenis* significantly overrepresented in early-stage lesions.

According to Pyakurel et al. (2025), healthy cattle could be distinguished from M2 and M4 groups based on skin biopsy analyses. *T. medium* occurred significantly more frequently in M4-stage lesions compared to M2. Their results from healthy skin biopsy samples showed the presence of *Treponema* spp., with quantitative real-time PCR detecting *T. medium* in 4 out of 25 samples (16%), *T. phagedenis* in 16/25 (64%), and *T. pedis* in 11/25 (44%). In our study, although skin biopsies were not analyzed, swab samples from the interdigital space were examined and compared between BDD-positive cows and healthy individuals. In the BDD-positive group, *T. medium* was detected in 40% of samples, *T. pedis* in 12%, and *T. brennaborense* in 4%. Among healthy cows, *T. medium* was present in 21.3% of cases, *T. pedis* in 15 samples (4.8%), and *T. brennaborense* in 1 sample (1.3%). Additionally, *T. phagedenis* subsp. *vaccae* and *T. socranskii* subsp. *buccale* were each detected in one and two samples, respectively (0.6%). Pyakurel et al. (2025) suggested that the higher bacterial diversity in clinical healthy skin decreases significantly in BDD lesions, often coinciding with an increased prevalence of *Treponema* spp. However, our limited detection of *T. phagedenis* subsp. *vaccae* and *T. socranskii* subsp. *buccale* in only three samples does not fully support this hypothesis. Nonetheless, the comparison of interdigital swab results indicates a higher presence of *T. medium* in the BDD-positive group. Interestingly, the detection rate of *T. brennaborense* in fecal samples was comparable between groups, as was the consistent absence of *T. medium* in fecal samples across both BDD-positive and healthy cows. Several studies have reported an increased abundance of *Treponema* species in BDD lesions (Beninger et al. 2018; Caddey and De Buck 2021). Similarly, Wong et al. (2024) observed a significant increase in both richness and relative abundance of *Treponema* spp. in BDD lesions compared to the skin microbiome of healthy animals. Alsaaod et al. (2019) also detected *Treponema* in clinically healthy cows, including *T. phagedenis* in 7 of 25 samples. While Dias et al. (2025) report that *T. phagedenis*, *T. medium*, and *T. pedis* do not persist on healthy foot skin, we detected these species in samples collected from clinically healthy cows. Although our cross-sectional data do not allow us to draw conclusions about the persistence of the bacteria, the presence of these *Treponema* spp. at one time point suggests that healthy skin may be transiently colonized.

Our results indicate the presence of *T. pedis* in 3 out of 25 interdigital swab samples from cows with BDD, compared to 15 out of 310 samples in the non-lesion (healthy) group. Pyakurel et al. (2025) reported a strong association between *T. pedis* abundance and M2-stage BDD lesions, as compared to non-lesion groups. In our study, however, the difference in *T. pedis* detection at the M2 stage was less pronounced than that observed for *T. medium*. Nevertheless, when examining surface swabs collected directly from M2 lesions, we confirmed a strong association, with *T. pedis* detected in 22 out of 25 samples. In contrast, the difference in *T. medium* detection between interdigital space swabs (23/25) and surface lesion swabs (9/25) was not as distinct. According to Wong et al. (2024), the microbial communities on the surface of active BDD lesions (M2 and M4.1) differ significantly from those on healthy skin (M0), though little to no difference was observed between the microbial profiles of M2 and M4.1 lesions. Similarly, Dias et al. (2024) found that cows without BDD lesions (M0) harbored all seven targeted bacterial species on the skin of the feet and other anatomical sites, with significantly higher bacterial counts in the presence of open lesions. In contrast, no significant differences were observed between other body skin sites, suggesting that compromised skin provides a more favorable environment for bacterial colonization. This hypothesis is supported by Wilson-Welder et al. (2015), who proposed that macro- and microabrasions caused by abrasive bedding materials may facilitate colonization by *Treponema* spp. and other BDD-associated pathogens. During our sampling, no visible skin damage was observed in the group of healthy cows; however, the possibility of microtrauma or subtle skin surface disruptions cannot be entirely excluded. Most studies documenting such lesions have been based on postmortem analyses, under controlled conditions that differ substantially from on-farm environments. For instance, McPherson et al. (2024) detected *Treponema* spp. in the deep dermal layer of two biopsies from healthy feet, in 6 of 21 superficial biopsies from BDD-affected feet, and in 6 of 19 deep-layer biopsies from the same animals. Such differences may be influenced by the sequencing technologies used, the number of samples analyzed, or the BDD lesion stages included (Krull et al. 2014). Moreover, microbial communities identified from swabs have been shown to differ from those obtained via biopsies (Duncan et al. 2021), likely due to environmental contamination of open wounds in live animals. Given the limitations of lesion biopsy sampling—especially its invasive nature and the challenge of repeated sampling—there is a growing need to validate less invasive techniques. Approaches such as swabbing or fine-needle aspiration are particularly promising in the context of modern, targeted molecular diagnostics.

The presence of *Treponema* spp. associated with BDD in fecal samples may originate from the gastrointestinal tract, but could also result from contamination via other biological secretions, direct contact between feces and BDD lesions, or environmental sources (Klitgaard et al. 2017). An intriguing observation by Potterton et al. (2011) suggests that cattle with more heavily soiled limbs tend to have fewer foot lesions, potentially indicating a protective role of dirt. This hypothesis is partially supported by Dias et al. (2024), who noted that while poor hygiene is associated with skin damage and an increased risk of BDD, it does not necessarily serve as a direct transmission route or reservoir for BDD-causing pathogens. In herds with endemic BDD, *T. phagedenis* and *T. medium* were simultaneously detected in most slurry samples but were absent from bedding material. Although six of the targeted *Treponema* species were detected in slurry and bedding, none of the seven investigated species were found in fecal samples. These results contrast with our findings, where, in cows with BDD lesions, *T. brennaborense* was detected in 28% and *T. pedis* in 8% of fecal samples. Among clinically healthy cows, *T. brennaborense* was found in 22.3% and *T. pedis* in 8.4% of fecal samples. *T. medium* was not detected in either group. These results suggest no significant differences in the presence of *T. brennaborense* and *T. pedis* in feces between BDD-positive and BDD-negative cows. Moreover, the relatively uniform distribution of these species across the herd, regardless of clinical status, may indicate that fecal shedding is not directly correlated with active lesion presence. It is important to acknowledge that the presence of nucleases in fecal matter may interfere with nucleic acid amplification by degrading DNA or inhibiting enzymatic reactions. Although we successfully detected *Treponema* spp. in fecal samples, their observed abundance was relatively low compared to previously reported levels. It is plausible that nuclease activity contributed to reduced amplification efficiency, potentially leading to false-negative results or underestimation of *Treponema* prevalence in feces.

Once introduced into a herd, bovine digital dermatitis (BDD) appears to be extremely difficult to eradicate, and complete elimination of its causative agents is even less likely (Grimm et al. 2025). In the event of an outbreak, the cornerstone of effective hoof health management lies in regular monitoring of BDD lesions and prompt primary veterinary treatment of cows exhibiting painful lesions. As highlighted by Gillespie et al. (2020), strict attention must also be paid to the disinfection of hoof trimming equipment, which plays a critical role in preventing the mechanical transmission of BDD-associated pathogens. Inter-herd transmission may also be facilitated by the use of external service providers. According to McPherson et al. (2024), veterinarians perform hoof care in approximately 45% of herds, while Yang et al. (2019) reported that hoof trimmers were involved in 9.9% of herds. These external contacts may inadvertently contribute to the spread of infection if proper biosecurity protocols are not consistently applied. Since the farm included in our study did not routinely implement footbaths, it is possible that the absence of this hygienic measure contributed to the transmission of bacteria to otherwise healthy feet. Although our study was not specifically designed to evaluate this association, regular footbaths may help reduce surface colonization and could be considered a potential preventive strategy (Döpfer et al. 2012). The clinical stage of BDD is a key factor in determining effective treatment strategies. Chronic lesions, in particular, are thought to act as persistent reservoirs of infection, complicating both therapeutic interventions and herd-level control efforts (Yang et al. 2020). Although only a single chronic lesion (M4 stage) was detected during our sampling period, the possibility of these lesions serving as reservoirs cannot be excluded. Recurrence of BDD is common. For example, Moreira et al. (2018) reported recurrence rates as high as 32% among dairy cows, underscoring the need for sustained and comprehensive management strategies aimed at minimizing lesion reoccurrence and disease persistence within affected herds.

The primary objective of most studies investigating bovine digital dermatitis (BDD), including ours, is to assess the presence and prevalence of the disease in dairy herds and to characterize the microbiota associated with both healthy skin and lesions in affected cattle. A secondary, yet equally important, aim is to identify potential herd-level risk factors that may influence the occurrence and persistence of BDD. Our study has several limitations. It was conducted on a single commercial dairy farm in which BDD was endemic, and standard herd management practices were employed, notably without the use of footbaths. As such, while the findings offer valuable insights, they may not fully capture the broader epidemiological context of BDD in Slovakia. To gain a more comprehensive understanding of the pathogenesis, etiology, and distribution of BDD, further research involving multiple herds from various geographic regions is necessary. It should also be noted that the results presented here may not be entirely representative of all Slovak dairy herds, particularly those with different management systems, environmental conditions, or biosecurity measures.

## Conclusions

This study confirmed the presence of *Treponema* species not only in lesions characteristic of bovine digital dermatitis (BDD) but also on the skin of clinically healthy cows and in fecal samples, albeit at lower prevalence. The results support the hypothesis that certain *Treponema* spp., particularly *T. medium* and *T. pedis*, are strongly associated with active BDD lesions, while others may transiently colonize healthy skin or originate from environmental contamination. The observed differences in microbial communities between lesion and non-lesion samples underscore the complex ecology of *Treponema* spp. within dairy herds (Nally et al. 2015). These findings highlight the importance of continued research involving larger sample sizes, multiple herds, and longitudinal study designs to better elucidate the transmission dynamics, persistence, and epidemiological roles of BDD-associated treponemes. Such data are essential for the development of more targeted and effective prevention and control strategies in dairy production systems.

## Data Availability

No datasets were generated or analysed during the current study.
